# Influence of Platelet Concentration on the Clinical Outcome of Platelet-Rich Plasma Injections in Knee Osteoarthritis

**DOI:** 10.1177/03635465241283463

**Published:** 2024-10-14

**Authors:** Angelo Boffa, Luca De Marziani, Luca Andriolo, Alessandro Di Martino, Iacopo Romandini, Stefano Zaffagnini, Giuseppe Filardo

**Affiliations:** * Clinica Ortopedica e Traumatologica 2, IRCCS Istituto Ortopedico Rizzoli, Bologna, Italy; ‡ Applied and Translational Research Center, IRCCS Istituto Ortopedico Rizzoli, Bologna, Italy; § Faculty of Biomedical Sciences, Università della Svizzera Italiana, Lugano, Switzerland; Investigation performed at IRCCS Istituto Ortopedico Rizzoli, Bologna, Italy

**Keywords:** PRP, platelets, concentration, knee, osteoarthritis

## Abstract

**Background:**

Platelet-rich plasma (PRP) is one of the most frequently used orthobiologic products for the injection treatment of patients affected by knee osteoarthritis (OA). Some preliminary evidence supports the influence of platelet concentration on patients’ clinical outcomes.

**Purpose:**

To analyze if platelet concentration can influence the safety and clinical efficacy of PRP injections for the treatment of patients with knee OA.

**Study Design:**

Cohort study; Level of evidence, 3.

**Methods:**

This study consisted of 253 patients with knee OA (142 men, 111 women; mean ± SD age, 54.8 ± 11.4 years; Kellgren-Lawrence grades 1-3) who were treated with 3 intra-articular injections of 5 mL of autologous leukocyte-rich or leukocyte-poor PRP. All patients were prospectively evaluated at baseline and at 2, 6, and 12 months. Patients were clinically assessed thorough the Knee injury and Osteoarthritis Outcome Score (KOOS) subscales and the International Knee Documentation Committee (IKDC) Subjective score. Platelet concentration was correlated with clinical outcome. Further analysis was performed by stratifying patients into 3 groups (homogeneous for OA severity) based on platelet concentration (high, medium, and low). All complications and adverse events were reported, as well as failures.

**Results:**

An overall statistically significant improvement in all clinical scores was documented from baseline to each follow-up evaluation. Platelet concentration positively correlated with clinical outcome. KOOS Pain improved more with higher platelet concentration at 2 months (*P* = .036; rho = 0.132), 6 months (*P* = .009; rho = 0.165), and 12 months (*P* = .014; rho = 0.155). The same trend was shown by the other KOOS subscales and by the IKDC Subjective score, as well as by the comparison of the groups of high-, medium-, and low-platelet PRP. The highest failure rate (15.0%) was found in the low-platelet group as compared with the medium-platelet group (3.3%) and the high-platelet group (3.3%). No differences were observed among the 3 groups in terms of adverse events.

**Conclusion:**

This study demonstrated that platelet concentration influences the clinical outcome of PRP injections in knee OA treatment. PRP with a higher platelet concentration provides a lower failure rate and higher clinical improvement as compared with PRP with a lower platelet concentration, with overall better results up to 12 months of follow-up in patients with knee OA.

Platelet-rich plasma (PRP) is one of the most frequently used orthobiologic products for the injective treatment of patients affected by knee osteoarthritis (OA).^14^ Platelets constitute a reservoir of growth factors, cytokines, chemokines, and many other bioactive molecules,^19,21,27,32^ which can modulate the intra-articular environment in osteoarthritic knees, with the potential of controlling inflammation, reducing symptoms, and modifying the disease progression.^8,18^ Besides some controversial results, the overall efficacy of PRP injection for patients with knee osteoarthritis (OA) is increasingly recognized, with several meta-analyses demonstrating better results as compared with saline or other injective treatments such as corticosteroids and hyaluronic acid.^3,18,28^ However, despite the promising results and the widespread clinical use, controversial aspects remain regarding the most suitable PRP composition.

PRP can be obtained with different preparation methods, which can yield products with different composition and properties.^34^ In particular, the presence of leukocytes and platelet concentration are the most debated aspects on PRP efficacy and are considered the main discriminants to distinguish PRP products.^1,16,23^ Despite some preclinical evidence suggesting the proinflammatory role of leukocytes in PRP and possible deleterious effects,^9^ recent high-level studies have demonstrated that the presence of leukocytes did not significantly affect the clinical results of PRP injections in clinical practice.^16^ Yet, the role of platelet concentration remains unclear. Preclinical studies suggested that platelets have a concentration-dependent effect, with high–platelet concentration PRP containing higher growth factor levels and inducing higher anabolic effects when compared with low–platelet concentration PRPs.^2,11^ Moreover, some preliminary evidence supports the influence of platelet concentration on patients’ clinical outcomes.^5,6,30^ However, although these findings are mainly derived from preliminary reports or indirect literature comparisons, large clinical studies directly investigating the role of platelet concentration are lacking.

The aim of this study was to analyze if platelet concentration can influence the safety and clinical efficacy of PRP injections for the treatment of patients with knee OA. The hypothesis was that PRP with higher platelet concentrations would provide better clinical outcomes in patients with knee OA.

## Methods

### Study Design

The present study is based on the analysis of a prospectively collected database of patients treated with intra-articular PRP injections for knee OA at the IRCCS Istituto Ortopedico Rizzoli (Bologna, Italy). The study was approved by the hospital ethics committee of the IRCCS Istituto Ortopedico Rizzoli (protocol 0014287), and patients were enrolled by orthopaedic surgeons between September 2016 and August 2022 in a research outpatient clinic focused on knee OA treatment. Informed consent was obtained from each patient for study participation. The following inclusion criteria were used for selection: male or female patients affected by unilateral symptomatic knee OA with a history of chronic pain or swelling (at least 4 months), age between 18 and 80 years, imaging findings of knee OA (Kellgren-Lawrence grades 1-3) evaluated by weightbearing anteroposterior and lateral radiographs, and failed results after at least 2 months of nonoperative treatment (eg, rest, physical therapy, anti-inflammatory and analgesic medications, and reduction of physical activity). The following exclusion criteria were applied for the selection: trauma or any kind of intra-articular injection therapy within 6 months or knee surgery within 12 months before treatment, major axial deviation (>5° of varus or valgus) evaluated by clinical examination with full-length leg radiographs if necessary, the presence of any concomitant knee lesion causing pain or swelling evaluated by clinical examination (eg, untreated knee instability, meniscal lesions occurring after trauma and assessed by magnetic resonance imaging [MRI], focal chondral or osteochondral defects requiring surgery), neoplasms, uncontrolled metabolic disorders, severe cardiovascular diseases, rheumatoid arthritis, inflammatory arthropathy, hematologic diseases, infections, immunodepression, anticoagulants or antiaggregant therapy, nonsteroidal anti-inflammatory drug use in the 5 days before blood harvest, and hemoglobin level <11 g/dL or platelet count <150,000/mm at blood harvest.

### Patient Characteristics

In total, 253 consecutive patients were enrolled according to the inclusion/exclusion criteria. There were 142 men and 111 women, with a mean ± SD age of 54.8 ± 11.4 years and a mean body mass index of 26.0 ± 4.2. Other patient characteristics are reported in Table 1. The PRP procedure consisted of 3 (1-week interval) intra-articular injections (without ultrasound guidance) of 5 mL of autologous PRP, which was activated with calcium gluconate, with a mean platelet concentration 4 times higher than baseline whole blood values, including PRP with and without leukocytes. PRP was produced via a laboratory method (no commercial PRP kit) or the CPunT preparation system (ELTEK Group) based on the institutional protocol available at the time of patient recruitment. PRP characteristics are reported in Table 2. All the procedures were performed by clinicians blinded to the leukocyte and platelet concentration of PRP. Each PRP injected was analyzed by the laboratory in terms of platelet and leukocyte concentrations. The mean of the 3 PRP values was used as the value of platelets and leukocytes injected during the treatment of each patient.

**Table 1:** Patient Characteristics

	No. or Mean ± SD (Range)
Sex: men/women	142/111
Age, y	54.8 ± 11.4 (21-75)
Body mass index	26.0 ± 4.2 (15.8-41.0)
Symptoms duration, mo	61.5 ± 65.9 (2-384)
Kellgren-Lawrence	
Grade 1	23
Grade 2	136
Grade 3	94

**Table 2:** PRP Characteristics^*a*^

	Whole Blood	PRP
Mean ± SD		
Platelets, 10^9^/L	244.1 ± 46.0	1043.0 ± 227.0
Erythrocytes, 10^9^/L	5.0 ± 0.9	0.3 ± 0.3
Leukocytes, 10^6^/L	6.3 ± 1.5	6.7 ± 6.2

a For each patient, whole blood and PRP concentrations were obtained from the mean of the 3 values obtained at each PRP treatment. PRP, platelet-rich plasma.

### Patient Evaluation

All patients were prospectively evaluated at baseline and then at 2, 6, and 12 months after the last injection. After enrollment in the study and at each follow-up visit, patients were clinically assessed thorough knee-specific patient-reported outcome measurements, including the International Knee Documentation Committee (IKDC) Subjective score and the Knee injury and Osteoarthritis Outcome Score (KOOS) subscales. Subjective clinical questionnaires were compiled by patients with the support of the clinician. All complications and adverse events were assessed and reported during follow-up visits, evaluating the safety of PRP injection treatments. Mild adverse events were defined as the presence of significant pain or swelling of the treated knee for at least 5 days as reported by patients, and severe adverse events were defined as any event that resulted in death, was life-threatening, or required hospitalization or interventions to prevent permanent impairment or damage. The treatment was deemed failed if the knee required a new injection or surgical procedure owing to persistent or worsening symptoms. For patients with failed results, the last clinical evaluation indicating the negative condition causing treatment failure was considered for follow-ups.

Platelet concentration was correlated with the clinical outcome, and to further investigate the influence of platelet concentration on the clinical effects of PRP injections, the included patients were stratified into 3 groups according to the concentration of platelets contained in the injected PRP based on the cutoff of 1,000,000 ± 20% platelets/µL^20^: low-platelet group, with a platelet concentration <800,000 platelets/µL; medium-platelet group, with a platelet concentration between 800,000 and 1,200,000 platelets/µL; and high-platelet group with a platelet concentration >1,200,000 platelets/µL.

### Statistical Analysis

All continuous data were expressed in terms of mean and standard deviation; categorical variables were expressed as proportions or percentages. The Shapiro-Wilk test was performed to test the normality of continuous variables. The Levene test was performed to test the homoscedasticity of continuous variables among groups. Pearson chi-square with an exact test was performed to investigate relationships between grouping variables. Analysis of variance repeated measures followed by a post hoc Sidak pairwise test were performed to assess the differences of the scores at the different follow-up times.

The analysis of variance test, followed by a post hoc Sidak test for pairwise comparisons, was performed to assess the between-group differences of continuous, normally distributed, and homoscedastic data; the Kruskal-Wallis nonparametric test, followed by a post hoc Dunn test for pairwise comparisons, was used otherwise. The Spearman rank correlation was used to assess correlations between the scores and the PRP characteristics.

For all tests, *P* < .05 was considered significant. All statistical analysis was performed with SPSS Version 19.0 (IBM Corp).

## Results

### Overall Clinical Results

An overall statistically significant improvement in all clinical scores was documented from baseline to each follow-up evaluation. The IKDC Subjective score significantly increased from the baseline level of 45.8 ± 15.9 to 57.3 ± 18.7 at 2 months, 59.7 ± 20.5 at 6 months, and 59.9 ± 21.5 at 12 months (all *P* < .0005 vs baseline). This improvement exceeded the minimal clinically important difference (MCID) of 8.5 at all follow-ups.^7^ The KOOS Pain score significantly increased from the baseline level of 64.0 ± 17.7 to 74.1 ± 18.4 at 2 months, 75.8 ± 19.5 at 6 months, and 75.3 ± 20.6 at 12 months (all *P* < .0005 vs baseline), exceeding the MCID of 9.1 at all follow-ups.^7^ Similar results were reported for the other KOOS subscales—Symptoms, Activities of Daily Living (ADL), Sport and Recreation, and Quality of Life (QOL)—overall exceeding the MCIDs of 8.2, 9.2, 11.6, and 10.3, respectively, as reported in Table 3. Severe adverse events were not reported, while mild adverse reactions, such as joint pain or swelling after 1 of the 3 injections, were reported in 42 patients (16.6%). In addition, the treatment of 13 patients (5.1%) was considered failed because they required additional knee treatments, such as knee injections (10 patients, 3.9%) or knee replacement (3 patients, 1.2%), during the follow-up period.

**Table 3:** Clinical Scores at Follow-up^*a*^

Score	Baseline	2 mo	6 mo	12 mo
IKDC Subjective	45.8 ± 15.9	57.3 ± 18.7	59.7 ± 20.5	59.9 ± 21.5
KOOS				
Pain	64.0 ± 17.7	74.1 ± 18.4	75.8 ± 19.5	75.3 ± 20.6
Symptoms	64.3 ± 17.5	73.4 ± 17.3	74.5 ± 19.5	74.8 ± 20.1
Activities of daily living	72.6 ± 19.5	81.5 ± 18.2	82.3 ± 19.0	81.4 ± 20.6
Sport and recreation	47.8 ± 19.9	57.9 ± 18.2	60.6 ± 24.4	60.4 ± 26.1
Quality of life	38.3 ± 18.5	51.7 ± 22.8	55.3 ± 24.8	55.7 ± 26.7

a Data are presented as mean ± SD. Statistically significant improvement (*P* < .05) occurred from baseline to every evaluated follow-up (2, 6, and 12 months). IKDC, International Knee Documentation Committee; KOOS, Knee injury and Osteoarthritis Outcome Score.

### Influence of Platelet Concentration

Platelet concentration was found to influence clinical improvement after PRP injections, with better clinical results in patients treated with PRP with a higher platelet concentration. In detail, the platelet concentration positively correlated with the improvement from baseline to 2 months in terms of IKDC Subjective score (*P* = .030; rho = 0.137), KOOS Pain (*P* = .036; rho = 0.132), KOOS Symptoms (*P* = .026; rho = 0.140), KOOS Sport/Recreation (*P* = .019; rho = 0.147), and KOOS QOL (*P* = .041; rho = 0.129). It positively correlated with improvement from baseline to 6 months in terms of IKDC Subjective score (*P* = .019; rho = 0.148) and KOOS Pain (*P* = .009; rho = 0.165). Finally, it positively correlated with improvement from baseline to 12 months in terms of KOOS Pain (*P* = .014; rho = 0.155), KOOS Symptoms (*P* = .036; rho = 0.132), KOOS Sport/Recreation (*P* = .048; rho = 0.124), KOOS ADL (*P* = .024; rho = 0.141), and KOOS QOL (*P* = .019; rho = 0.147).

**Figure 1.**
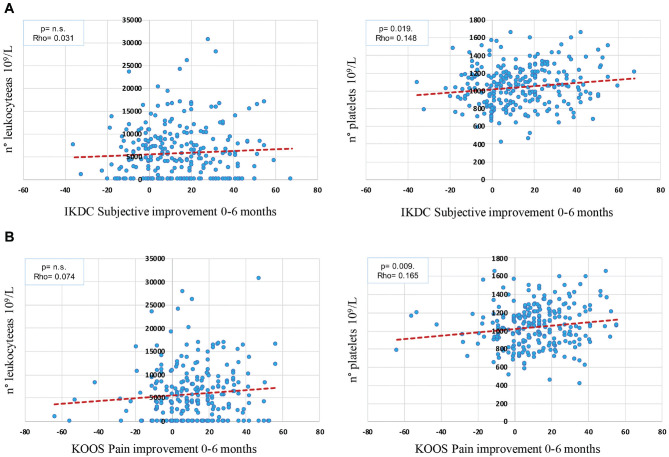
Score improvement from baseline to 6 months of follow-up and correlation with PRP number of leukocytes (10^9^/L; not significant) and platelets (10^9^/L): (A) IKDC Subjective (*P* = .019; rho = 0.148) and (B) KOOS Pain (*P* = .009; rho = 0.165). IKDC, International Knee Documentation Committee; KOOS, Knee injury and Osteoarthritis Outcome Score; PRP, platelet-rich plasma.

Leukocyte concentration was investigated to understand the possible influence on clinical improvement after PRP injections, with no correlation found for all clinical scores at all follow-up evaluations, as well as for adverse events (Figure 1).

### Clinical Results Based on Platelet Concentration

Among the included patients, 40 were treated with PRP with a platelet concentration <800,000 platelets/µL (low-platelet group), 153 with a platelet concentration between 800,000 and 1,200,000 platelets/µL (medium-platelet group), and 60 with a platelet concentration >1,200,000 platelets/µL (high-platelet group). The 3 groups were homogeneous for all baseline demographic and clinical characteristics, except body mass index, which was slightly lower (1.6) in the low-platelet group. The characteristics of the 3 groups are reported in the Table 4.

**Table 4:** Patient Characteristics of the 3 Groups Based on PRP Platelet Concentration^*a*^

	Platelet Concentration, No. or Mean ± SD			
	Low (n = 40)	Medium (n = 153)	High (n = 60)	*P* Value
Sex: men/women	20/20	84/69	38/22	NS
Age, y	53.3 ± 13.0	55.1 ± 11.2	55.0 ± 10.9	NS
Body mass index	24.6 ± 3.4	26.2 ± 4.6	26.4 ± 3.4	.041
Symptoms duration, mo	80.7 ± 93.7	63.7 ± 65.7	43.3 ± 31.8	NS
Kellgren-Lawrence				NS
Grade 1	1	17	5	
Grade 2	22	80	34	
Grade 3	17	56	21	
PRP				
Platelets, 10^9^/L	706.5 ± 87.7	1011.2 ± 111.8	1345.2 ± 120.4	<.0005
Leukocytes, 10^6^/L	4.8 ± 4.6	5.5 ± 5.2	7.5 ± 7.2	NS

a NS, not significant; PRP, platelet-rich plasma.

No differences were observed among the 3 groups in terms of adverse events. A different failure rate was found among the 3 groups (*P* = .009). The highest failure rate (15.0%, 6 patients) was found in the low-platelet group as compared with the medium-platelet group (3.3%, 5 patients) and the high-platelet group (3.3%, 2 patients).

An overall improvement from baseline to all follow-ups was found in the 3 groups. However, even though the medium- and high-platelet groups significantly improved (*P* < .0005) from baseline to all follow-ups for all scores (IKDC Subjective score and KOOS subscales), the low-platelet group did not show a significant improvement in all scores, with no improvement in KOOS Pain and KOOS ADL. Moreover, the low-platelet group showed significant improvement in terms of KOOS Symptoms (*P* = .022) and KOOS Sport/Recreation (*P* = .004) only at 6 months, without showing significant improvements at 2 and 12 months of follow-up. More details on the clinical scores of the 3 groups are reported in Table 5.

**Table 5:** Clinical Scores of the 3 Groups Based on Platelet Concentration^*a*^

Outcome: Group	Baseline	2 mo	6 mo	12 mo	*P* Value^*b*^
**IKDC Subjective**					
Low	43.15 ± 17.2	49.6 ± 21.7^*c*^	53.5 ± 24.8^*c*^	53.5 ± 24.2	.004
Medium	46.45 ± 15.6	57.9 ± 18.4	59.0 ± 19.3	59.8 ± 20.6	<.0005
High	45.86 ± 15.9	60.5 ± 15.7^*c*^	65.6 ± 19.3^*c*^	64.5 ± 21.1	<.0005
**KOOS**					
Pain					
Low	63.1 ± 18.5	68.5 ± 21.9	69.9 ± 22.2^*c*^	68.2 ± 25.1	NS
Medium	64.4 ± 17.7	73.9 ± 18.4	74.8 ± 19.1^*d*^	74.9 ± 20.5	<.0005
High	63.3 ± 17.7	78.3 ± 14.7	82.4 ± 16.7^*c*,*d*^	80.8 ± 16.0	<.0005
Symptoms					
Low	60.2 ± 17.4	62.5 ± 20.7^*c*,*e*^	68.5 ± 21.6^*c*^	67.1 ± 23.0^*c*^	.017
Medium	64.6 ± 17.2	74.3 ± 15.9^*e*^	74.5 ± 18.2	74.3 ± 19.5^*d*^	<.0005
High	66.3 ± 18.2	78.4 ± 15.6^*c*^	78.6 ± 20.5^*c*^	81.4 ± 17.9^*c*,*d*^	<.0005
ADL					
Low	74.7 ± 19.4	78.3 ± 21.5	80.5 ± 22.3	76.6 ± 25.8	NS
Medium	71.9 ± 19.9	80.8 ± 18.7	80.6 ± 19.2^*d*^	80.6 ± 20.3	<.0005
High	73.0 ± 19.0	85.2 ± 13.6	87.8 ± 15.3^*d*^	86.5 ± 16.4	<.0005
Sport and recreation					
Low	42.7 ± 19.7	49.2 ± 23.9^*c*^	56.9 ± 29.2	50.1 ± 27.9^*c*^	.005
Medium	48.7 ± 19.6	58.2 ± 21.4	59.5 ± 22.5	60.7 ± 25.2	<.0005
High	48.8 ± 20.3	63.0 ± 22.6^*c*^	65.7 ± 25.3	66.3 ± 25.4^*c*^	<.0005
Quality of life					
Low	35.6 ± 19.4	44.0 ± 22.3^*c*^	49.0 ± 24.4^*c*^	48.6 ± 28.3^*c*^	.002
Medium	38.6 ± 18.0	51.1 ± 22.4	54.0 ± 23.6^*d*^	54.4 ± 25.6^*d*^	<.0005
High	39.2 ± 19.3	58.3 ± 22.9^*c*^	62.7 ± 26.4^*c*,*d*^	64.0 ± 26.4^*c*,*d*^	<.0005

a ADL, activities of daily living; IKDC, International Knee Documentation Committee; KOOS, Knee injury and Osteoarthritis Outcome Score; NS, not significant;.

b Analysis of variance.

c *P* < .05 in favor of high- vs low-platelet group.

d *P* < .05 in favor of high- vs medium-platelet group.

e *P* < .05 in favor of medium- vs low-platelet group.

The comparative analysis among the 3 groups showed better results for patients of the high-platelet group. In particular, the high-platelet group showed a significantly higher improvement from baseline when compared with the low-platelet group in terms of IKDC Subjective score at 2 months (14.7 ± 13.4 vs 6.5 ± 14.3; *P* = .007); KOOS Pain at 2 months (14.9 ± 13.8 vs 5.3 ± 16.6; *P* = .007), 6 months (19.1 ± 16.2 vs 6.74 ± 18.2; *P* = .002), and 12 months (17.4 ± 17.8 vs 5.1 ± 22.8; *P* = .016); KOOS Symptoms at 2 months (12.1 ± 14.1 vs 2.3 ± 14.7; *P* = .003); KOOS ADL at 2 months (12.2 ± 13.5 vs 3.6 ± 14.5; *P* = .008), 6 months (14.8 ± 15.5 vs 5.8 ± 16.2; *P* = .034), and 12 months (13.5 ± 17.3 vs 1.9 ± 20.9; *P* = .010); KOOS Sport/Recreation at 2 months (14.2 ± 21.0 vs 6.5 ± 18.6; *P* = .039); and KOOS QOL (19.1 ± 19.1 vs 8.4 ± 18.1; *P* = .025). Moreover, the high-platelet group showed significantly higher improvement from baseline when compared with the medium-platelet group in terms of IKDC Subjective score at 6 months (19.7 ± 18.9 vs 12.5 ± 17.8; *P* = .035), KOOS Pain at 6 months (19.1 ± 16.2 vs 10.4 ± 18.5; *P* = .007), and KOOS QOL at 12 months (24.8± 26.5 vs 15.7 ± 23.1; *P* = .049). Finally, the medium-platelet group showed significantly higher improvement from baseline when compared with the low-platelet group in terms of KOOS Symptoms at 2 months (9.7 ± 15.8 vs 2.3 ± 14.7; *P* = .025).

## Discussion

The main finding of this study was that platelet concentration influences the clinical outcome of PRP injections for the treatment of patients affected by knee OA. PRP with a higher platelet concentration provided a lower failure rate and higher clinical improvement when compared with PRP with a lower platelet concentration. This sheds new light on the debated topic of the factors influencing PRP results and on the indications toward the most suitable PRP preparation for knee OA.

Intra-articular PRP injections have gained increasing interest in the last 2 decades in the management of mild to moderate cases of knee OA,^8,15^ showing safety and efficacy in several high-level trials and meta-analyses, with superiority over placebo and other injection treatments regardless of the platelet and leukocyte concentration.^18^ In this context, there is a change in the position of some major international societies,^24,29^ with the most recent consensus of the European Society of Sports Traumatology, Knee Surgery and Arthroscopy even concluding that PRP injections are not only a valid treatment option for knee OA but can be considered a possible first-line injectable treatment option for nonoperative management of knee OA, mainly for Kellgren-Lawrence grades 1 to 3.^24^ Yet, the consensus group was not able to provide clear indications on the most suitable platelet concentration because of the lack of data in the literature to investigate the correlation between the number of platelets and the clinical response to PRP. In this light, platelet concentration represents one of the most debated and less investigated aspects regarding PRP efficacy, with only preliminary data and indirect literature comparisons speculating on the role of platelet concentration.^5,6,23,30^ A recent small randomized controlled trial (RCT) compared 25 patients treated with a high-dose PRP (8 mL) with 25 patients treated with a low-dose PRP (4 mL), reporting overall clinical trends and results in favor of the high-dose PRP group.^30^ These authors concluded that PRP with approximately twice the number of platelets can offer better clinical results in patients with knee OA. A systematic review of the literature found that studies reporting statistically significant positive outcomes used PRP with a higher platelet number as compared with studies not finding positive results.^6^ Similarly, a recent meta-analysis compared indirectly the clinical results offered by PRP with different platelet concentrations in patients with knee OA by analyzing 18 RCTs.^5^ The authors found that studies with PRP formulations with higher platelet concentrations demonstrated superior pain relief and more durable functional improvement as compared with studies on PRP with lower platelet concentrations.

The current study is the largest study directly evaluating the influence of platelet concentration on PRP clinical efficacy. The analysis of >250 patients with knee OA, treated with PRP injections in the same institution by the same orthopaedic team, demonstrated that platelet concentration influences the clinical outcome. A positive correlation was found between platelet concentration and clinical scores at all follow-up evaluations. Moreover, the stratification of patients into 3 groups based on platelet concentration provided further interesting results. In fact, patients with a platelet concentration >1,200,000 platelets/µL showed the best clinical outcomes, while patients with a platelet concentration <800,000 exhibited less satisfactory clinical outcomes. PRP with higher platelet concentrations also provided a lower failure rate than PRP with lower platelet concentrations (3% vs 15%). Furthermore, although PRP with higher platelet concentrations provided significant improvement in all the evaluated clinical scores, the improvements obtained after PRP with lower platelet concentrations were only partial. The results of this study shed new light on the PRP field and could explain some controversial literature findings on the potential of this orthobiologic approach for patients with knee OA.

The lack of PRP efficacy in patients with knee OA demonstrated by some studies could be related to the use of PRP with low platelet concentrations. In a recent RCT on 288 patients aged >50 years with low-grade knee OA treated with PRP or saline solution, Bennell et al^4^ reported no clinical superiority of PRP injections over placebo up to 12 months of follow-up and no improvement in terms of MRI evaluation. However, the PRP used in that study had a relative low platelet concentration as compared with the current study, in line with the poor benefits obtained by the low-platelet PRP injections demonstrated in this study. Yet, the positive results obtained after PRP injections in other studies could be because of the use of PRP with higher platelet concentrations. A larger RCT on PRP injections for knee OA was performed by Chu et al^13^ on 610 patients treated with intra-articular injections of PRP with a high platelet concentration. This study showed the superiority of PRP over placebo in terms of (1) clinical improvement up to 60 months of follow-up, (2) synovial biomarker profile at 6 months with lower values of interleukin 1 and tumor necrosis factor α, and (3) MRI findings with a lower decrease of the tibiofemoral cartilage volume >60 months. In this light, platelet concentration may represent a crucial factor and should always be carefully considered when interpreting the results of PRP injections. To this regard, this study documented that PRP with a platelet concentration >1,200,000 platelets/µL provided higher clinical effects on different subscales over PRP with platelet concentrations ranging between 800,000 and 1,200,000 platelets/µL (medium group) and <800,000 platelets/µL (lower group). This result suggests that higher concentrations of platelets might lead to superior clinical outcomes. However, caution must be exercised, taking into account the in vitro studies on PRP, which suggest that excessively high concentrations of platelets can have deleterious effects.^35^ Therefore, future studies will need to identify the optimal platelet concentration to optimize PRP use as injectable treatment of knee OA.

Beyond the platelet concentration, another debated topic of the PRP field is the role of leukocytes. In vitro studies have suggested that leukocytes can influence PRP properties, owing to the release of catabolic and proinflammatory molecules, which could be detrimental from a clinical point of view.^2,9^ The current study analyzed the possible influence of leukocytes on the clinical outcome offered by PRP injections. Leukocyte concentration did not affect the clinical response to PRP treatment in this study on a large series of patients prospectively evaluated up to 12 months of follow-up. These results are consistent with those obtained in a recent double-blind RCT conducted by Di Martino et al^16^ on 192 patients with knee OA. These authors compared the use of 2 similar PRPs, with the only discriminant being leukocyte concentration. They demonstrated that leukocytes did not influence the clinical outcome of PRP injection treatment, with comparable results between leukocyte-rich and leukocyte-poor PRP in terms of clinical improvement, adverse events, and treatment failures. Even though a lot of attention has been placed in the last decade on the presence of leukocytes, current clinical evidence indicates that the leukocyte effects shown in vitro may not be translated into clinically perceptible differences after PRP injections. Yet, the focus should be shifted toward the role of the platelet concentration, and this study confirmed the importance of this PRP factor in terms of clinical efficacy.

Platelets are the key players in determining PRP properties, thanks to their ability to release numerous growth factors and anti-inflammatory cytokines, which can affect the OA joint environment.^2,17,25^ For this reason, clinical trials on PRP injections should always report the platelet concentration of the product used to allow readers to fully interpret their results. However, a review by Chahla et al^12^ highlighted how only 10% of the studies provided complete information on the PRP preparation protocol and only 16% reported quantitative parameters on the composition of the final PRP product. Different PRP classification systems have been proposed in recent years^23,26,31^ underlining the importance of documenting PRP composition, and it is crucial that future studies adhere to these recommendations to advance the field by providing further evidence on the most suitable PRP preparation.

This study documents a large cohort of patients with PRP quantification of platelets and leukocytes, which was followed prospectively up to 12 months, but it presents some limitations. This study is not an RCT directly comparing 2 types of PRP with different platelet concentrations, and future trials should confirm the results with a higher-level design. Even though the study groups demonstrated homogeneity in terms of demographic characteristics, it cannot be excluded that other factors may contribute to the results, thus biasing the conclusions on PRP concentrations. The content of growth factors and other specific characteristics of the injected PRP were not evaluated, as suggested by current guidelines, and future studies should investigate if the growth factor content could influence the treatment efficacy. Patients were evaluated with subjective clinical scores, while imaging findings were not investigated. Nevertheless, this aspect has been previously evaluated, and no significant differences have been reported in OA severity progression at short-term follow-up in terms of Kellgren-Lawrence grade in radiographs or cartilage thickness at MRI assessment.^10,22,33^ Despite these limitations, this study sheds some light on the controversies revolving around PRP composites. Although the presence of leukocytes has not been shown to influence clinical outcome, platelet concentration plays a role in determining the patient's clinical response. Still, platelet and leukocyte concentrations are not the only parameters potentially capable of modifying the characteristics and efficacy of PRP. This is a composite product in which many variables can play a role, including the preparation method, the activation modality, and the number of and time between injections. All of these should be investigated to optimize PRP preparation and use. Moreover, identifying the type of patient and the disease stage that could benefit the most from the injections would further improve the clinical potential when applying this biologic treatment. In this complex scenario, the results of this study are of clinical relevance. The use of PRP with high platelet concentrations seems advantageous over PRP with low platelet concentrations, although high-level RCTs directly comparing PRP products with different concentrations are needed to confirm these findings and optimize the use of PRP in patients affected by knee OA.

## Conclusion

This study demonstrated that platelet concentration influences the clinical outcome of PRP injections in knee OA treatment. PRP with a higher platelet concentration provides a lower failure rate and higher clinical improvement than PRP with a lower platelet concentration, with overall better results up to 12 months of follow-up in patients with knee OA.
